# Approach of Multiple Endocrine Neoplasia Type 1 (MEN1) Syndrome–Related Skin Tumors

**DOI:** 10.3390/diagnostics12112768

**Published:** 2022-11-12

**Authors:** Livia-Cristiana Băicoianu-Nițescu, Ana-Maria Gheorghe, Mara Carsote, Mihai Cristian Dumitrascu, Florica Sandru

**Affiliations:** 1 Department of Dermatology, Elias University Emergency Hospital, 011461 Bucharest, Romania; 2 Department of Endocrinology, C.I. Parhon National Institute of Endocrinology, 011863 Bucharest, Romania; 3 Department of Endocrinology, C. Davila University of Medicine and Pharmacy & C.I. Parhon National Institute of Endocrinology, 011683 Bucharest, Romania; 4 Department of Obstetrics and Gynaecology, C. Davila University of Medicine and Pharmacy & University Emergency Hospital, 050474 Bucharest, Romania; 5 Department of Dermatology, C. Davila University of Medicine and Pharmacy & Elias University Emergency Hospital, 011368 Bucharest, Romania

**Keywords:** multiple endocrine tumors, MEN1, angiofibromas, collagenomas, lipomas, melanoma, endocrine, gene, skin

## Abstract

Non-endocrine findings in patients with MEN1 (multiple endocrine neoplasia) syndrome also include skin lesions, especially tumor-type lesions. This is a narrative review of the English-language medical literature including original studies concerning MEN1 and dermatological issues (apart from dermatologic features of each endocrine tumor/neuroendocrine neoplasia), identified through a PubMed-based search (based on clinical relevance, with no timeline restriction or concern regarding the level of statistical significance). We identified 27 original studies involving clinical presentation of patients with MEN1 and cutaneous tumors; eight other original studies that also included the genetic background; and four additional original studies were included. The largest cohorts were from studies in Italy (N = 145 individuals), Spain (N = 90), the United States (N = 48 and N = 32), and Japan (N = 28). The age of patients varied from 18 to 76 years, with the majority of individuals in their forties. The most common cutaneous tumors are angiofibromas (AF), collagenomas (CG), and lipomas (L). Other lesions are atypical nevi, basocellular carcinoma, squamous cell carcinoma, acrochordons, papillomatosis confluens et reticularis, gingival papules, and cutaneous T-cell lymphoma of the eyelid. Non-tumor aspects are confetti-like hypopigmentation, café-au-lait macules, and gingival papules. *MEN1* gene, respective menin involvement has also been found in melanomas, but the association with MEN1 remains debatable. Typically, cutaneous tumors (AF, CG, and L) are benign and are surgically treated only for cosmetic reasons. Some of them are reported as first presentation. Even though skin lesions are not pathognomonic, recognizing them plays an important role in early identification of MEN1 patients. Whether a subgroup of MEN1 subjects is prone to developing these types of cutaneous lesions and how they influence MEN1 evolution is still an open issue.

## 1. Introduction

Multiple endocrine neoplasia type 1 syndrome (MEN1) is a rare autosomal dominant disorder that leads to pituitary, parathyroid, and gastropancreatic neuroendocrine tumors (NET) [1]. MEN1 is also known as Wermer syndrome, considering that an association of endocrine tumors with an autosomal dominant pattern of inheritance was first described by Wermer in 1954 [2]. MEN1 has a prevalence between 1 in 20,000 and 1 in 40,000 and equal estimated prevalence for both sexes [3,4]. However, a higher prevalence in females, between 55% and 57%, has been described by some authors [3,4]. No racial, ethnic, or geographical differences in prevalence have been observed [5]. An increased prevalence has occasionally been observed in certain areas due to a founder effect [6,7].

The cause is represented by loss of function germline mutations in the *MEN1* gene [1,8]. *MEN1* is a tumor suppressor gene on chromosome 11q13 that encodes menin, a ubiquitous, predominantly nuclear protein in non-dividing cells, with no intrinsic enzymatic activity [9,10,11]. Menin is involved in regulating gene transcription, genome stability, and cell proliferation by interacting with various proteins, either as a co-repressor or co-activator [10,12,13,14,15,16,17]. Through interactions with chromatin-associated protein complexes and regulation of non-coding RNAs, menin is involved in the epigenetic control of gene expression [10,12,13,14,15,16,17]. According to Knudson’s ‘two-hit hypothesis’, in order for a tumor to develop, a secondary event, such as large chromosomal deletions, is necessary to occur and cause biallelic inactivation of the MEN1 gene [1,18,19]. In accordance with this theory, tumor DNA displays loss of heterozygosity at chromosome 11q13 in over 90% of cases [1,9,20,21,22]. Other secondary events, accounting for the remaining 10% of cases where loss of heterozygosity is absent, include point mutations or a small insertion or deletion in the splice sites or coding region of the *MEN1* gene [1].

More than 80% of affected individuals develop clinical manifestations by the age of 50 [1]. The estimated penetrance by the age of 40 years reaches 90% for parathyroid tumors, 40% for gastrinoma, and 30–40% for pituitary NETs [3,23]. Although it is a highly penetrant syndrome, the types of tumors an affected individual might develop cannot be predicted due to a lack of genotype–phenotype correlation [24,25,26,27]. Based on guideline level, individuals can be diagnosed with MEN1 if they develop two of the three main MEN1-related tumors (pituitary, parathyroid, and pancreatic neuroendocrine tumors); if they present with one MEN1-associated tumor and are a first-degree relative of a patient diagnosed with MEN1; or if, despite being asymptomatic, a mutation of the *MEN1* gene is confirmed [1]. MEN1 patients present lactotroph tumors (the most frequent pituitary NETs associated with MEN1, followed by somatotroph tumors) in addition to primary hyperparathyroidism (which typically appears around the age of 20–25 years and is more frequently accompanied by nephrolithiasis than non-MEN hyperparathyroidism) [24,25,26,27,28,29,30]. Between 30 and 80% of patients develop pancreatic NETs. More than half of them are gastrinomas that cause Zollinger–Ellison syndrome (ZES) and up to 30% are insulinomas [1]. In contrast with adults, gastrinomas are a rare, but aggressive, occurrence in pediatric patients [1,3]. Apart from the main NETs, MEN1 is associated with other tumors such as NETs of the thymus, lung, and ovary, adrenal cortical tumors including adrenal carcinoma, pheochromocytoma, and, also, meningiomas [1,31,32]. Thyroid tumors also occur in 25% of patients, but the association may not be causal [1]. An increased risk of breast cancer in female patients with MEN1 has also been described, but a causal link has not been definitely established [4,31,33,34].

The most common cutaneous tumors found in MEN1 are angiofibromas, collagenomas, and lipomas [35,36,37]. Typically, these are benign, and are usually surgically treated only for cosmetic reasons [35,38]. *MEN1* gene-respective menin involvement has also been described in melanomas, but the association with the syndrome remains debatable [39]. Skin lesions are not limited to tumors, but also include confetti-like hypopigmentation, café-au-lait macules, and gingival papules [35,36]. Even though skin lesions are not pathognomonic, recognizing them plays an important role in early identification of MEN1 patients [35,38].

A multidisciplinary approach to MEN1 treatment is essential due to significant morbidity and an important reduction of life expectancy, requiring adequate screening and frequent follow-ups [40,41]. Genetic testing is an important tool, both for confirming the diagnosis and identifying affected family members [42]. In patients affected by MEN1, 71% of deaths are caused by MEN1, most importantly by thymus and duodenopancreatic NETs, including non-secreting pancreatic tumors, which harbor a malignant potential and are often clinically silent [43]. Patients with MEN1 are associated with more severe outcome than non-MEN1 patients with similar tumors; therefore, early differentiation between MEN1 and sporadic tumors is extremely important [1]. In addition, MEN1 diagnosis negatively impacts the quality of life of patients and may be accompanied by feelings of anxiety and depression, especially in patients with a high burden of disease and treatment. These patients may require psychological support [44,45]. Being in a clinical surveillance and therapy program and being provided adequate information by the clinicians was found to increase the satisfaction of patients, by assuring early treatment of emerging tumors [46]. Any delay in diagnosis can have harmful consequences both for patients and their family members. Therefore, early recognition and timely diagnosis of index cases and affected family members are crucial for proper management. Increasing awareness among physicians of multiple specialties is essential in reducing negative outcomes [47]. Despite the rarity of MEN1, its significant morbidity and mortality makes early recognition crucial for MEN1 patients. Dermatological issues associated with MEN1, when observed by aware physicians, may raise suspicion and call for further investigation, resulting in early diagnosis for several patients.

Our purpose is to provide practical insight concerning MEN1-related skin profile, following specific dermatological tumors in patients with this syndrome.

## 2. Methods

This is a narrative review of the English-language medical literature in which we included 40 original papers concerning MEN1 and dermatological tumors, identified through a PubMed-based search, with the following keywords: “dermatological”, “skin”, “cutaneous”, “angiofibroma”, “collagenoma”, “lipoma”, “melanoma”, in different associations with “MEN1”, “multiple endocrine neoplasia”. The papers were included based on clinical relevance specifically addressing the dermatological issues in patients confirmed with the syndrome, without timeline restriction, and without concern for the level of statistical significance. The subsections are organized following various types of dermatological tumors that we identified through the research. Exclusion criteria were represented by specific dermatologic signature of each endocrine tumor and/or neuroendocrine neoplasia as recognized components of MEN1.

## 3. MEN1 and Skin Tumor Profile

The most common cutaneous tumors found in patients with MEN1 were angiofibromas, collagenomas, and lipomas. Other cutaneous tumors found were melanomas, atypical nevi, basocellular carcinoma, squamous cell carcinoma, acrochordons, papillomatosis confluens et reticularis, gingival papules, and cutaneous T-cell lymphoma of the eyelid, but this category is not sustained by well-designed clinical studies and genetic workup in most cases, thus the association might be incidental (Table 1) [31,39,48,49,50,51,52,53,54,55,56,57,58,59].

The frequencies of most frequent three cutaneous tumors typically associated with MEN1 syndrome, as identified by the largest three studies we found, are summarized in Table 2 [31,48,49,53,57,69].

Angiofibromas are the most frequent cutaneous tumors in patients with MEN1, followed by collagenoma and lipoma. The findings of Darling et al. and Asgharian et al. are similar [48,53]. Marini et al., however, found lipomas with a higher prevalence than all other skin lesions [69].

In order to determine the impact of dermatological assessment of patients with pancreatic endocrine tumors in diagnosing MEN1, Asgharian et al. investigated the presence of cutaneous tumors in patients with ZES, sporadic or associated with MEN1. Out of 110 patients with ZES, 48 patients had MEN1, while 62 subjects had sporadic pancreatic endocrine tumors. The patients were assessed for the presence of angiofibromas, lipomas, collagenomas, and other dermatological tumors. Angiofibromas were present in 64% of the MEN1 patients and only 8% of the individuals with sporadic ZES. More than three angiofibromas were found in 50% of the MEN1 patients, while none of the patients with sporadic ZES met this criterion. Collagenomas were also more frequent in patients with MEN1, where they were found in 62% of the cases, in comparison with 5% of patients with ZES. Other skin tumors found in MEN1 patients were lipomas, basal cell carcinoma, melanoma, and acrochordons. However, the study found no statistically significant difference in terms of frequency. Several possible criteria for identifying patients with MEN1, based on the presence of angiofibromas, collagenomas, lipomas, and combinations, were analyzed. The criterion with the highest combination of sensitivity and specificity was more than three angiofibromas or any collagenoma, with a sensitivity of 75% and specificity of 95% [53].

Darling et al. performed an observational 3-year study, on 32 patients previously diagnosed with MEN1 who did not have personal or family history of tuberous sclerosis. The patients were evaluated for the presence of cutaneous lesions. The findings included cutaneous tumors (angiofibromas, collagenomas, and lipomas), gingival papules, and confetti-like hypopigmented macules. The most frequent cutaneous lesion observed was angiofibroma. Multiple facial angiofibromas affected 28 (88%) of the patients. Five or more angiofibromas were found in half of the patients. The second most frequent finding was collagenoma, with 23 (72%) patients being affected by collagenomas. Lipomas were observed in 11 (34%) patients. Multiple gingival papules were also found in two (6%) patients [48].

Marini et al. conducted a retrospective study of 145 patients with MEN1 syndrome and 20 asymptomatic gene carriers in the Tuscany region of Italy. The majority of patients, 106 (64.2%), were female, while 59 (35.8%) of them were males. In terms of cutaneous lesions, lipomas were the most frequent finding that were confirmed in 37 (25.52%) patients. Out of the 37 patients, 17 presented with single lipomas and 20 with multiple lipomatosis. Angiofibromas were found in nine (6.21%) patients. Other cutaneous tumors found were three fibromas and four angiomas (but not angiofibromas). Moreover, in seven cases (4.83%), lipomas represented the first clinical manifestation of MEN1 syndrome (before endocrine tumors) [69].

### 3.1. Angiofibromas

Angiofibromas are the most frequent skin manifestation associated with MEN1 syndrome, according to most studies [48,69,73]. They are benign cutaneous vascular tumors that contain thin-walled blood vessels, stellate and spindled cells, and collagen bundles, and they present as pink or red shiny surfaced papules [74,75]. Apart from MEN1, multiple facial angiofibromas can be found in Birt-Hogg-Dubé syndrome and tuberous sclerosis as well [75]. These tumors used to be considered pathognomonic for tuberous sclerosis; however, over the years, it has been shown that they are the most common cutaneous manifestation of MEN1 syndrome, as well [76,77]. In MEN1, the most frequent location is facial, mainly the upper lip, in contrast with tuberous sclerosis, where this location is spared [76,78]. Angiofibromas were the most frequent cutaneous tumor observed and were found in 18 studies according to our research [48,49,50,52,53,54,55,56,57,59,62,63,64,66,67,68,69,70]. Angiofibromas in MEN1 patients are reported with a frequency between 11% and 88% on studies of different sample size (varying from 145 to 5 patients) [48,49,69] (Table 2). Yet, we mention another study on 90 patients which did not report any angiofibromas despite identifying other skin tumors (31). Facial angiofibromas is recognized as the most frequent location in MEN1, but thoracic site was also described [54].

Differences between the Japanese population and Caucasians were observed by Sakurai et al. [52]. Sakurai et al. found a lower frequency of angiofibromas in MEN1 patients in the Japanese population than Darling et al. found in Caucasians, despite similar other group characteristics, including age and frequency of endocrine tumors. In addition, angiofibromas were fewer in the Japanese population. While Darling et al. found only multiple angiofibromas, Sakurai et al. noted that 7 out of 28 patients presented with a single angiofibroma [48,52]. Table 3 introduced the three studies we identified with most details concerning angiofibromas in MEN1, comparing the findings of Sakurai, Darling, and Asgharian et al. [48,52,53].

In terms of impact on diagnosis, Asgharian et al. found that more than three angiofibromas or any collagenoma identifies MEN1 patients with a sensitivity of 75% and specificity of 95% in patients with Zollinger–Ellison syndrome [53]. Even though angiofibromas are most often part of a genodermatosis, one case report presents an asymptomatic female patient with more than 100 facial angiofibromas unassociated with a genetic syndrome [79]. Similarly, Hunter et al. presented four patients with multiple facial angiofibromas who did not suffer MEN1 or tuberous sclerosis [80]. Despite not being incidental, angiofibromas in MEN1 are still incompletely described in terms of correlations with clinical endocrine phenotype and genetic background.

### 3.2. Collagenomas

Collagenomas are hamartomatous proliferations of the connective tissue that usually present as well-circumscribed dome-shaped, skin-colored, or hypopigmented nodules or papules. They have a firm consistency and are usually found on the upper trunk and neck, often distributed in a symmetrical pattern. Unlike angiofibromas, the face is a rare site for collagenomas [53,81,82,83]. Collagenomas were observed in 11 studies based on our analysis in MEN1 subjects [48,49,53,55,58,59,61,62,63,65,72]. Their prevalence was between 40% and 72%. Even though collagenomas are frequent, the largest two studies did not find any collagenomas in the patients they evaluated [31,69] (Table 2). Collagenomas were most often accompanied by other cutaneous tumors. They were, however, the single cutaneous tumors in four case reports [58,61,65,72].

### 3.3. Lipomas

Lipomas are benign, well-capsulated, subcutaneous tumors with origin in the adipocyte. They have a high prevalence in the general population and are the most common fat-containing tumor. Lipomas can also be found in association with genetic syndromes including MEN1, Birt-Hogg-Dubé, and Cowden Syndrome. In MEN1 syndrome, lipomas are less frequent than other cutaneous tumors and are often solitary [53,83,84,85,86]. Lipomas were observed in 11 studies according to our research criteria [31,48,49,53,54,57,59,60,69,70,71]. The prevalence of lipomas, as reported in studies with a number of patients between 5 and 145, was between 17% and 34% [31,48,49,53,57,69].

Of note, as opposite to angiofibromas and collagenomas, each of the most extended studies reported cases with this type of skin tumor (Table 2). However, despite finding statistical significance for the association between angiofibromas, collagenomas, and MEN1 syndrome, Asgharian et al. did not find a statistically significant difference in the prevalence of lipomas in MEN1 syndrome (17%) compared to sporadic ZES (16%). The presence of lipomas did not correlate with the age of the patients or with the duration of the disease [53]. Lipomas presented either as solitary or multiple lesions [31,49,53,54,57,59,60]. In terms of size, there was one case, reported by Fushimi et al., of a giant lipoma with a cervical location [71].

Similar to angiofibromas, lipomas have been reported as the first manifestation of MEN1 syndrome in two studies [59,69]. Marini et al. found lipomas as the first clinical manifestation in seven patients [69]. Another case with cutaneous manifestations as the first findings was reported by Zeller et al. The patient first developed lipomas as a teenager, approximately two decades earlier than the first endocrine manifestation. In his case, lipomas were multiple, on the neck and extremities [59].

Among the mutations associated with lipomas, we mention: W436R, 579delG in exon 3, c.812_820del, p.Gly271_Leu273del, c.1613delA [49,54,70,71]. Lipomas without clearly identified mutations were also found in association with endocrine tumors. Schulte et al. assessed the *MEN1* gene in two patients with endocrine tumors and lipomas, but found no mutations of the same gene. One of the patients had multiple lipomas in association with a pituitary NET, while the other patient had multiple lipomas accompanied by recurrent goiter. The patients were not further assessed for other components of MEN syndrome [87].

### 3.4. Genetic Considerations in Skin Tumors Associated with MEN1

A possible genetic mechanism of angiofibromas, collagenomas, and lipomas of patients with MEN1 syndrome is represented by the loss of heterozygosity of the *MEN1* gene [49]. In support of this theory are the studies of Pack et al., respective of Dong et al. [49,88]. Dong et al. assessed 13 patients with MEN1, three of whom had angiofibromas and two associated lipomas. Genetic analysis was employed to search for loss of heterozygosity, considered as a 90% decrease of one allele compared to constitutional DNA, using 10 polymorphic markers of the *MEN1* gene at 11q13. There was no loss of heterozygosity found in the informative loci of the angiofibromas in this study. Potential explanation behind the absence of loss of heterozygosity includes the possibility of a different mechanism involved in the occurrence of angiofibromas or the inability to detect loss of heterozygosity due to either contamination or the limitations of the method used, which could not identify intragenic or point mutations. Concerning the lipomas, loss of heterozygosity was found in one of the two examined lipomas [88]. On the other hand, Pack et al. found allelic deletion of the *MEN1* gene in all facial angiofibromas of the five patients, in three collagenomas and one lipoma. In contrast with angiofibromas in MEN1, the angiofibroma of a patient with tuberous sclerosis did not exhibit allelic deletion. Other cutaneous tumors found by Pack et al. in subjects with MEN1 were a melanocytic nevus and an acrochordon. Allelic deletion of the *MEN1* gene was not present in these tumors, thus they seem incidental [49].

The perspective of genetic testing in patients with these three types of cutaneous lesions is represented by the focused research of skin lesions that, among others, might be a clue for MEN1 or the dermatologic assessment in individuals coming from families that harbor *MEN* mutations. Boni et al. studied 19 sporadic angiofibromas unassociated with clinically manifested MEN1 syndrome or tuberous sclerosis, and found mutations of the *MEN1* gene in two tumors, meaning an A→T transition at nucleotide 517 in exon 2, and a transversion of GG→AA at nucleotide 1184–5 in exon 8. None of the 19 angiofibromas had loss of heterozygosity of the *MEN1* gene [89]. Cavaco et al. performed a mutational analysis of 26 MEN1 patients who were members of six families. Facial angiofibromas were present in two individuals, with 1539delG and del (Exon 7–3′ untranslated region) mutations [90]. Loss of heterozygosity was also found by Morelli et al. in one visceral lipoma out of six MEN1-associated lipomas [91].

Table 4 summarizes the genetic mutations of patients with MEN1 syndrome and the most frequent cutaneous tumors (angiofibroma, lipoma, collagenoma) [49,52,54,56,58,60,65,66,67,70,71,90].

### 3.5. Melanoma: Any Connection with MEN1?

Melanoma has a rare occurrence in MEN1 patients. However, there are some case reports that present this type of skin cancer in MEN1. *MEN1* gene was studied in relation to this malignancy. The findings include the role of *MEN1* gene in tumor suppression of melanoma and the role of menin in inhibiting melanoma cell migration and proliferation. However, other studies were unable to find MEN1 mutations in melanomas [51,53,57]. Nord et al. assessed seven cases of melanoma in patients with MEN1. All patients suffered from primary hyperparathyroidism. Other tumors found were thymus carcinoid, bronchial carcinoid, and ependymoma. The genetic mutations found were: 360dupGT, 776delC, W220X, 1657insC, IVS 2–3 (splice), linked mutation. Loss of heterozygosity was not screened for in the melanomas associated with MEN1. The study also analyzed 19 metastatic sporadic melanomas searching for loss of heterozygosity in the chromosome 11q13 region. Six out of 19 tumors confirmed loss of heterozygosity [51]. Vidal et al. described the presence of melanocyte-derivate cancer in one of the nine patients assessed (11.1%), without an increased number of nevi. The tumor was diagnosed during the routine following of MEN1 syndrome [57]. Fang et al. found that the *MEN1* gene acts as a tumor suppressor gene in melanoma. This gene stimulates the transcription of genes involved in homologous-directed DNA repair. Moreover, *MEN1* expression was reduced in nine out of eleven melanoma samples that were analyzed [92]. Gao et al.’s findings support the role of the *MEN1* gene in tumor suppression in melanoma. They found that menin inhibited melanoma cell migration and proliferation, both in vitro and in vivo, through several signaling pathways [93]. Lazova et al. performed an exome-wide sequencing study on melanoma and two other benign cutaneous lesions—Spizoid nevi and conventional nevi. Genetic similarities between Spizoid nevi and melanoma were found, including mutations of the *MEN1* gene [94]. Bóni et al., however, found no evidence of the *MEN1* gene’s involvement in the development and growth of melanoma. The study analyzed possible mutations of the *MEN1* gene in 23 cutaneous melanoma and 17 melanoma metastases. No *MEN1* gene mutations or loss of heterozygosity were found in any of the cases [95].

Even though angiofibromas are the most frequent cutaneous tumors in MEN1, differential diagnosis with other cutaneous tumors should be taken into account. For instance, we mention one such case. Brown et al. report a patient diagnosed with MEN1 as a teenager with surgically treated primary hyperparathyroidism, who presented for a red, telangiectatic lesion on the helix, initially considered to be an angiofibroma. The patient also had multiple facial angiofibromas. Biopsy revealed that the tumor was an amelanotic melanoma, with histological traits of angiofibroma in the vicinity of the tumor [64].

As far as we currently know, the presence of a melanoma in a MEN1 patient is not part of the typical picture and it is probable that other genetic constellations or implications are responsible for this association. Whether a subgroup of MEN1 individuals is prone to develop dermatological tumors is still an open issue. For example, we mention the report of Baldauf et al. concerning possible unrelated multiple skin masses in a patient with MEN1. The 70-year-old male patient, known with MEN1 for 10 years, was diagnosed with three different cutaneous tumors, one of benign origin (papillomatosis confluens et reticularis), and two malignant tumors (a melanoma and a squamous cell carcinoma). Genetic analysis of these two malignancies did not reveal loss of heterozygosity with respect to *MEN1* gene. Despite the presence of three different dermatologic tumors, the patient did not present more common MEN1 cutaneous manifestations such as angiofibroma, collagenoma, or lipoma [39].

### 3.6. Other Skin Tumors in MEN1 Patients

Apart from the most frequent cutaneous tumors, as mentioned (angiofibroma, collagenoma, and lipoma), a variety of different skin tumors have been described in MEN1 individuals; yet, the genetic confirmation and the statistical significance to strongly sustain a relationship between these lesions and endocrine syndromes is to be determined. We mention gingival papules (two papers), melanocytic nevus (one article), basal cell carcinoma (two papers), squamous cell carcinoma (two papers), acrochordon (two reports), papillomatosis confluens et reticularis (one case), trichodiscomas (one paper), and corneal xanthogranuloma (one article) [31,39,48,49,53,59,68,70].

Febrero et al. showed a case of an aggressive cutaneous T-cell lymphoma of the eyelid [31]. Whether MEN1 pro-tumor status is prone to abnormal hematologic proliferations is still under debate. Fibrofolliculomas, trichodiscomas, and acrochordons are typical cutaneous findings of Birt-Hogg-Dubé syndrome, a disease caused by loss of heterozygosity of the *FLCN* gene, which increases the risk of renal tumors and pneumothoraces [96,97]. However, trichodiscomas and acrochordons have been reported in patients with MEN1, without Birt-Hogg-Dubé syndrome [49,68]. Therefore, even though trichodiscomas and acrochordons are typical findings in Birt-Hogg-Dubé syndrome, differential diagnoses with alternative causes, such as MEN1, might be useful to take into consideration in some cases in order to provide proper diagnosis and management.

Alkatan et al. presented a case of bilateral corneal xanthogranuloma, a benign tumor that usually involves the skin or the eye, in a patient with MEN1 syndrome [98]. However, we did not find other reported cases of xanthogranulomas in patients with MEN1 syndrome. Gingival papules are oral fibromas that may potentially occur both in MEN1 and other genetic syndromes (such as tuberous sclerosis, Birt-Hogg-Dubé syndrome, and Cowden syndrome), underlying the importance of differential diagnosis between MEN1 and these syndromes when faced with cutaneous and mucosal tumors in addition to other tumors [48,59,99,100].

## 4. Discussion

### 4.1. Identifying MEN1 Patients Starting from Skin Tumors

In order to provide adequate diagnosis and early treatment of possible tumors underlying syndromes with endocrine tumors and/or NETs, individuals with specific cutaneous lesions should be further evaluated for MEN1, even when they are asymptomatic or without a known family history of MEN1 [63]. However, the level of statistical relevance of a larger scale is currently very low. There are a few published cases with cutaneous tumors (angiofibromas, collagenomas, and lipomas) as the first manifestation of MEN1 syndrome. In support of this aspect, we mention four case reports and one retrospective study, a total of 11 patients who were diagnosed with MEN1 after first presenting for mentioned cutaneous lesions [56,58,62,63,69]. Sakurai et al. reported a 38-year-old female patient with angiofibromas as the first manifestation of MEN1 syndrome [56]. Witchel et al. introduced a case of an 18-year-old female who was referred to endocrine evaluation after the identification of more than 70 collagenomas [58]. Roman et al. presented a 28-year-old male without a known family history of MEN1, who was first admitted for skin lesions. The patient was asymptomatic from an endocrine point of view and had normal laboratory tests. The biopsy confirmed the lesions were facial angiofibromas and a truncal collagenoma. Due to this combination of lesions, the patient underwent genetic testing for MEN1 syndrome and was positive [63]. As mentioned, Marini et al. found that seven out of 37 patients with lipomas associated with MEN1 had lipomas as the first manifestation [69]. In addition, two case reports included individuals who developed cutaneous tumors 5, respective 20 years prior to the actual MEN1 diagnosis [59,67]. The presence of angiofibromas is more frequent in patients with MEN1 syndrome than seen in subjects with sporadic ZES, as proved by Asgharian et al. In order to ensure an adequate screening and management of MEN1 syndrome, the differentiation between sporadic and syndromic pancreatic NETs and of other NETs with different sites is crucial, which is why dermatological screening could be a useful tool [53]. Of note, the importance of early diagnosis in MEN1 is due to high disease-related burden, and skin lesions might represent a useful hallmark. Awareness is the key factor, noting that the index of clinical suspicion is low. Regardless, MEN1 is already identified on a certain patient at the level of carrier or confirmed endocrine tumors/NETs.

### 4.2. Cherry Angiomas: From Acromegaly to MEN1

Belonging to the category of (skin) angiomas, cherry angiomas represents a distinct skin tumor apart from angiofibromas and have been associated with acromegaly, either sporadic or syndromic. Cherry angiomas are benign tumors caused by the proliferation of endothelial cells and are composed of tiny vascular channels originating from post-capillary venules in the upper part of the dermis [101,102,103]. Macroscopically, they present as red, small papules, and are most frequently located on the trunk and extremities [101,104,105]. Despite the pathogenesis not being entirely known, it has been suggested that high levels of insulin-like growth factor-1 (IGF-1) may play a role in the development and growth of cherry angiomas, making them present in association with a somatotropinoma [101,106]. Usually asymptomatic, cherry angiomas can potentially become a certain reason for concern from a cosmetic point of view. Several therapeutic approaches have been suggested including light-based devices such as Nd:YAG laser, intense pulsed light, and pulsed dye laser, as well as other non-laser options including sclerotherapy, cryotherapy, or electrotherapy [104,107,108]. Even though acromegaly represents an infrequent part of MEN1, with a penetrance of 10% due to its severe impact, the diagnosis of such skin findings should not be overlooked [1,109,110,111,112].

### 4.3. Skin Health Surveillance in Patients with MEN1

Depending on the type of dermatologic lesions, after first identification of mentioned tumors, genetic and endocrine workup might be recommended, unless the patient is already under periodic check-up for MEN1. After adequate identification, benign tumors are removed depending on site, local signs, suspected malignancy, and patient’s choice (for instance, cosmetic effects). Tumors with expected benign behavior as angiofibromas should be carefully examined, taking into consideration that sometimes they may be similar to more severe conditions as was the case of an amelanotic melanoma of the helix presented by Brown et al. [64]. Other non-melanoma cutaneous cancers, such as basal cell carcinoma, squamous cell carcinoma, or T-cell lymphoma have also been observed in MEN1, but the level of statistical significance is extremely low, thus, most probably, the association was incidental [31,53,59]. A patient with MEN1 syndrome may associate malignant cutaneous tumors, with or without a clear genetic connection, and this scenario should not be overlooked, but we cannot routinely recommend a certain protocol of dermatologic assessment and re-assessment depending on what we know so far. Moreover, integrating the dermatological issues to a general panel of a MEN1 patient evaluation should take into consideration an already complicated panel of surveillance due to traditional endocrine and neuroendocrine components and it should not supplementarily impact the overall quality of life in such individuals [25,38,113,114].

### 4.4. Cutaneous Lesions in MEN1: How Deep Should We Look?

Overall, we identified 27 original studies involving clinical presentation of patients with MEN1 syndrome and cutaneous tumors [31,39,48,49,50,51,52,53,54,55,56,57,58,59,60,61,62,63,64,65,66,67,68,69,70,71,72]. We also mentioned eight original studies involving the genetics of these types of skin tumors [88,89,90,91,92,93,94,95]. Four additional original studies were also introduced: two case reports of multiple cutaneous tumors unassociated with genodermatosis [79,80], one case report of an ocular tumor [98], and one case report with unconfirmed MEN1 syndrome [87]. The largest cohorts involving MEN1 syndrome and cutaneous tumors were of 145 (Italy), 90 (Spain), 48 and 32 (United States), and 28 individuals (Japan) [31,48,52,53,69]. Patients’ age varied from 18 to 76 years, with most patients being in their forties [31,39,48,49,50,51,52,53,54,55,56,57,58,59].

We did not limit the research to the pediatric population, but no such specific study was identified, as expected based on typical MEN1-associated clinical presentation [1]. Of course, the level of statistical relevance varies from cohorts to case reports, but we decided not to restrain the research, knowing that MEN1 syndrome may associate unusual pathological entities which should be assessed in order to find out if they are accidental on an individual confirmed with MEN1, are caused by genetic background of the syndrome, or are due to some hormonal anomalies induced by the associated endocrine tumors and/or NETs, as seen by IGF-1 excess [101,106].

Early identification of MEN1 is extremely important for screening and management in order to reduce mortality and morbidity. Cutaneous tumors such as angiofibroma, collagenoma, and lipoma are common findings and they particularly raise a MEN1 suspicion, needing to be differentiated from sporadic presentations, especially in patients with special features, as suggested by Asgharian et al. (more than three angiofibromas or any collagenoma) [53]. These tumors might be the earliest sign of MEN1, before endocrine complications are clinically relevant. We still do not know enough with respect to particular endocrine configuration concerning the subgroup of MEN1 individuals with skin findings and whether the relationship is in terms of skin issues influencing the endocrine evolution or quite the other way around. From a dermatologic perspective, differential diagnosis of angiofibromas, collagenomas, and lipomas should include other genetic syndromes (mostly genodermatoses like Birt-Hogg-Dubé, tuberous sclerosis, and Cowden Syndrome since skin is a strong player) in addition to different clinical endocrine and non-endocrine entities due to multiple gene anomalies [115,116,117,118,119,120]. It is also worth mentioning that sometimes, even though a number of cutaneous tumors typically associated with genodermatosis are present in the same patient, the occurrence might be coincidental according to current knowledge.

The variety and frequency of cutaneous tumors in patients with MEN1 syndrome call for careful skin check-ups, not only for angiofibromas, collagenomas, and lipomas, but also for melanoma [64]. However, the melanoma–MEN1 correlation due to common genetic background is debatable; also, a higher prevalence of this most aggressive skin cancer in MEN1 when compared to the general population has not been proved. In MEN1 patients, this malignancy is either a possible consequence of the *MEN1* gene defects, with loss of heterozygosity, or due to other gene and epigenetic anomalies (non-*MEN1*), and/or related to environmental factors such as persistent exposure to sunlight, etc. [120,121,122,123,124,125]. Therefore, close examination of skin lesions in patients with MEN1 might prove useful. Despite potential menin involvement in the development of melanoma, germline *MEN1* mutations are not prone to this malignancy, as, for instance, has been found with other genes such as *CDKN2A* [93,126,127]. Other hormonal dysfunctions involving growth factors, growth hormone–IGF-1 axes, or estrogens/estrogen receptor status have been reported in melanoma without a clear connection to endocrine tumors underlying MEN1 [128,129,130,131,132,133]. Larger clinical studies on MEN1 and potential skin involvement are necessary, including longitudinal data and comparison with patients without dermatological issues. Whether genetic, epigenetic, and/or hormonal anomalies in MEN1 are contributors to solid dermatologic tumors is still a very interesting, but open chapter.

Finally, the present topic of following the skin tumors in MEN1 represents a fraction of a more complex picture that includes cutaneous aspects in association with various tumor-related hormonal issues as seen in acromegaly, primary hyperparathyroidism, or similar findings in pancreatic NETs in addition to heterogeneous anomalies in MEN2 syndrome (involving both MEN2A and MEN2B type, such as dermal hyperneury, cutaneous lichen amyloidosis, sclerotic fibromas, atypical rash, macular amyloidosis, etc.), but also, clinical expression of carcinoid syndrome caused by gastroenteropancreatic NETs or metastatic medullary thyroid cancer [133,134,135,136,137,138,139,140,141]. A good dermatological—endocrinological collaboration is required to benefit our patients.

## 5. Conclusions

To our knowledge, this type of dermatologic approach with respect to an endocrine entity as MEN1 is among the rarest in the literature. Awareness regarding cutaneous tumors associated with this syndrome is beneficial for patients by preventing diagnosis delay and aiding to distinguish between genetic and sporadic cases. Most common skin findings are angiofibromas, collagenomas, and lipomas. Some cases indicate these lesions as first manifestation of MEN1. Even though skin solid tumors are not pathognomonic, recognizing them seems useful in the overall complicated picture of MEN1. Whether a subgroup of MEN1 subjects is prone to develop these types of cutaneous lesions, if their presence represents a certain risk factor or a surrogate of a certain endocrine condition and what their influence may be on MEN1 evolution is still an open issue.

## Figures and Tables

**Table 1:** Cutaneous findings in patients with MEN1 syndrome. Please see references [31,39,48,49,50,51,52,53,54,55,56,57,58,59].

Year/ Reference Number/ First Author	Type of Study	Studied Population/ Number of Patients	Age	Findings			Observations
				Endocrine	Skin	Others	
1997 [48] Darling	Case series	32 patients		hyperparathyroidism (100%), pancreatic NETs (50%), pituitary NETs (44%)	angiofibromas (88%), collagenomas (72%), lipomas (34%), multiple gingival papules (6%)		
1998 [49] Pack	Case series	5 patients: 2 (40%) males and 3 (60%) females	16 years		angiofibroma		
			42 years		angiofibromas		
			46 years		Collagenomas, melanocytic nevus		
			56 years		angiofibromas		
			69 years		angiofibroma, collagenoma and lipoma, acrochordon		
1999 [50] Hoang-Xuan	Case report	One male patient	42 years	hypercalcemia, pituitary microadenoma	angiofibroma		
2000 [51] Nord	Case series	7 patients: 5 (71%) males and 2 (29%) females	34 years	hyperparathyroidism, pancreatic NET	melanoma		
			30 years	hyperparathyroidism, pancreatic NET	melanoma		
			42 years	hyperparathyroidism	melanoma	thymic carcinoid	
			51 years	hyperparathyroidism	melanoma	bronchial carcinoid	
			68 years	hyperparathyroidism	melanoma	ependymoma	
			45 years	hyperparathyroidism, pancreatic NET	melanoma		
			54 years	hyperparathyroidism, pancreatic NET, pituitary NET	melanoma		
2000 [52] Sakurai	Case series	28 (27 MEN1 patients and one asymptomatic gene carrier): 14 males and 14 females	43 ± 17 years	hyperparathyroidism (96%), pituitary NETs (43%), pancreatic NETs (50%)	angiofibromas in 12 out of 28 patients (42.85%)		
2004 [53] Asgharian	Prospective study	48 patients with MEN1 and Zollinger–Ellison syndrome: 17 (35% male) and 31 (65%) female	48.6 ± 1.8 years	gastropancreatic NETs (Zollinger-Ellison syndrome), hyperparathyroidism (98%), pituitary NETs (38%)	angiofibromas (64%), collagenomas (62%), lipomas (17%), atypical nevi, basal cell carcinoma, squamous cell carcinoma, melanoma, skin tags, gingival hyperplasia		
2006 [54] Nuzzo	Case report	One male patient	51 years	hyperparathyroidism, pancreatic NET, pituitary NET (lactotroph), nonfunctioning adrenal adenoma	abdominal lipomas, facial and thoracic angiofibromas		Three of the five children of the patient were also affected. His son was also affected by multiple thoracic angiofibromamas.
2007 [55] Xia	Case report	One male patient	32 years	primary hyperparathyroidism, pituitary NET, pancreatic NET	collagenomas, angiofibromas		The patient associated rapid growing collagenomas shortly after pancreatic surgery. The patient had family history of MEN1 (mother and grandmother).
2008 [56] Sakurai	Case report	One female patient	38 years	no endocrine tumors at the time of diagnosis	facial angiofibroma		first manifestation
2008 [57] Vidal	Case series	9 patients: 4 (45%) males and 5 (55%) females	43.4 years (at the time of the study)	hyperparathyroidism (100%), pancreatic NETs (66%), pituitary NETs (44%)	angiofibromas (22.2%), lipomas (33.3%), melanomas (11.1%)	atypical thymic carcinoid (1 patient)	There were no collagenomas observed. All lipomas were multiple.
2009 [58] Witchel	Case report	One female patient	18 years	hyperparathyroidism	more than 70 collagenomas		
2009 [59] Zeller	Case report	One male patient	45 years	primary hyperparathyroidism, pituitary NET (lactotroph), gastrinoma, bilateral adrenal hyperplasia	multiple angiofibromas, collagenomas, fibrolipomas, gingival papules, fifty acrochordons		
2009 [39] Baldauf	Case report	One male patient	70 years	hyperparathyroidism, pancreatic NET	melanoma, papillomatosis confluens et reticularis, squamous cell carcinoma		No loss of heterozygosity; no other cutaneous manifestations
2011 [60] Rusconi	Case report	One female patient	23 years	pituitary NET, parathyroid hyperplasia	subcutaneous lipoma (spindle cell lipoma)		The patient was negative at mutation screening of MEN1 exons 2 to 10
2012 [61] Furtado	Case report	One male patient	35 years	pituitary NET, primary hyperparathyroidism, pancreatic gastrinoma	abdominal collagenomas		
2012 [62] Simi	Case report	One male patient	41 years	pituitary NET, parathyroid adenoma, pancreatic NET	collagenomas, seborrheic keratosis or angiofibroma		
2014 [63] Roman	Case report	One male patient	28 years	No endocrine tumors at the time of diagnosis	facial angiofibromas, truncal collagenoma		Incidental skin lesions led to MEN1 diagnosis.
2015 [64] Brown	Case report	One male patient	In his 20s	hyperparathyroidism	angiofibromas, amelanotic melanoma		The patient had significant sun exposure. He developed an amelanotic melanoma with histologic features of angiofibroma in the proximity of the tumor.
2015 [65] Perez	Case report	One male patient	45 years	pancreatic, parathyroid and pituitary NETs	multiple progressive collagenomas		
2016 [66] Okada	Case report	One male patient	44 years	primary hyperparathyroidism, pancreatic NETs, non-functional adrenal cortical adenoma, pheochromocytoma, pituitary NET	fibroma	neurofibroma	
2017 [67] Kaiwar	Case report	One female patient	76 years	pancreatic NET	facial angiofibromas	lymphoma	The patient had a family history of MEN1 in children. She was asymptomatic and was diagnosed following genetic screening. The pancreatic tumor was found on MRI. The skin lesions appeared at an old age, 4–5 years prior to diagnosis.
2017 [68] Yeh	Case report	One male patient	47 years	parathyroid adenomas, hyperthyroidism, pheochromocytoma, gastrinoma	angiofibromas, multiple trichodiscomas		The patient was negative for FLCN gene mutation
2018 [69] Marini	Retrospective epidemiological, clinical and genetic analysis	145 clinically affected and 20 asymptomatic mutation carriers: 59 (35.8%) males and 106 (64.2%) females	31.8 ± 13.5 years	139 (95.86%) primary hyperparathyroidism, 86 (59.31%) gastroenteropancreatic NETs, 75 (51.72%) pituitary NETs	37 (25.52%) lipomas, 9 (6.21%) angiofibromas, 3 fibromas, 4 angiomas		
2020 [70] Radman	Case report	One male patient	57 years	pancreatic gastrinoma, parathyroid hyperplasia	fibromas, lipomas, basocellular carcinoma	pulmonary NET grade 2, fibromyxoid sarcoma	
2021 [71] Fushimi	Case report	One male patient	28 years	pituitary NET, primary hyperparathyroidism, multiple abnormal fatty deposits in the pancreas	a giant cervical lipoma		The patient’s lipoma was considered very large in size.
2021 [31] Febrero	Retrospective analysis	90 patients: 50 (56%) males and 40 (44%) females		hyperparathyroidism (95%), pancreatic NETs (53%), pituitary NETs (40%), adrenal tumors (33%), nodular thyroid disease (10%), thyroid papillary carcinoma (1 patient), ovarian mucinous cystadenoma (1 patient)	lipomas (22%), cutaneous T-cell lymphoma of the eyelid	thymic tumors (2 patients), breast cancer (2 patients)	No collagenomas or angiofibromas.
2022 [72] Ranaweerage	Case report	One female patient	23 years	pituitary NET (lactotroph), primary hyperparathyroidism, insulinoma	multiple collagenomas on the chest and abdominal wall		PCOS

Abbreviations: NET = neuroendocrine tumor; PCOS = polycystic ovarian syndrome.

**Table 2:** Prevalence of cutaneous tumors in MEN1 patients as reported by largest studies in terms of number of patients diagnosed with the syndrome. Please see references [31,48,49,53,57,69].

Year/ Reference Number/ First Author	Number of Patients with MEN1	Angiofibromas/ Angiomas/ Fibromas, No. Patients (%)	Collagenomas, No. Patients (%)	Lipomas, No. Patients (%)
2018 [69] Marini	145	16 (11%)	0	37 (25.52%)
2021 [31] Febrero	90	0	0	20 (22%)
2004 [53] Asgharian	48	31 (64%)	30 (62%)	8 (17%)
1997 [48] Darling	32	28 (88%)	23 (72%)	11 (34%)
2008 [57] Vidal	9	2 (22.2%)	0	3 (33.3%)
1998 [49] Pack	5	4 (80%)	2 (40%)	1 (20%)

Abbreviations: No. = number of patients; MEN1 = multiple endocrine neoplasia.

**Table 3:** Analysis of angiofibromas in Caucasians [48], Japanese [52], and United States citizens diagnosed with MEN1 [53].

Year/ Reference Number/ First Author	1997 [48] Darling	2000 [52] Sakurai	2004 [53] Asgharian
Number of patients	32	28 (27 patients with familial MEN1 and 1 asymptomatic gene carrier)	48
Age of patients	39 ± 14 years	43 ± 17 years	48.6 ± 1.8 years
Frequency of angiofibromas	88% (28/32)	43% (12/28)	64% (31/48)
Frequency of patients with more than 10 angiofibromas	41% (13/32)	7% (2/28)	NA
Number of patients with a single angiofibroma	0	7	NA
Frequency of hyperparathyroidism	100%	96%	98%
Frequency of pituitary NETs	44%	43%	38%
Frequency of pancreas NETs	50%	50%	Only MEN1 patients with pancreatic endocrine tumors were selected

Abbreviations: MEN1 = multiple endocrine neoplasia; NET = neuroendocrine tumor.

**Table 4:** Summary of the genetic mutations of patients with MEN1 syndrome and the most frequent cutaneous tumors (angiofibroma, lipoma, collagenoma) [49,52,54,56,58,60,65,66,67,70,71,90].

Year/ Reference Number/ First Author	Genetic Mutations		
	Angiofibroma	Collagenoma	Lipoma
1998 [49] Pack	R460X, Y323X, Q260X, W436R	713delG, W436R	W436R
2000 [52] Sakurai	359del4, K119del, Q166X, 621del9, 1422insA, 1473del5, 1606del29, 1657insC	NA	NA
2006 [54] Nuzzo	Heterozygote frameshift 579delG in exon 3	NA	Heterozygote frameshift 579delG in exon 3
2008 [56] Sakurai	c.511_519del	NA	NA
2009 [58] Witchel	NA	C>A substitution in exon 4 of the MEN1 gene (predicted to generate a nonsense mutation)	NA
2011 [60] Rusconi	NA	NA	heterozygous deletion on chromosome band 11q13.1 that contained all of the MEN1 gene and the 50 end of the adjacent MAP4K2 gene
2015 [65] Perez	NA	c.265delC	NA
2016 [66] Okada	g.249_252delGTCT	NA	NA
2017 [67] Kaiwar	c.1A>G	NA	NA
2020 [70] Radman	heterozygous mutation c.812_820del, p.Gly271_Leu273del	NA	heterozygous mutation c.812_820del, p.Gly271_Leu273del
2021 [71] Fushimi	NA	NA	frameshift c.1613delA
2002 [90] Cavaco	NA	NA	1539delG, del (Exon 7–3′ untranslated region)

Abbreviations: MEN1 = multiple endocrine neoplasia; NA = not available.

## Abbreviations

IGF-1	insulin-like growth factor-1
MEN1	multiple endocrine neoplasia type 1
NET	neuroendocrine tumor
ZES	Zollinger–Ellison syndrome
